# Encapsulation Efficiency of Electrosprayed Glucose Oxidase Capsules: Effect of the Drying Technique

**DOI:** 10.3390/polym17040488

**Published:** 2025-02-13

**Authors:** Minerva Rentería-Ortega, María de Jesús Perea-Flores, Alberto Peña-Barrientos, Rigoberto Barrios-Francisco, Liliana Edith Rojas-Candelas, Georgina Calderón-Domínguez

**Affiliations:** 1 Tecnológico Nacional de México/Tecnológico de Estudios Superiores de San Felipe del Progreso, San Felipe del Progreso 50640, Mexico; minerva.ro@sfelipeprogreso.tecnm.mx (M.R.-O.); rigoberto.bf@sfelipeprogreso.tecnm.mx (R.B.-F.); lilianae.rc@sfelipeprogreso.tecnm.mx (L.E.R.-C.); 2 Centro de Nanociencias y Micro y Nanotecnologías, Instituto Politécnico Nacional, Ciudad de México 07738, Mexico; mpereaf@ipn.mx (M.d.J.P.-F.); apenab@ipn.mx (A.P.-B.); 3 Departamento de Ingeniería Bioquímica, Escuela Nacional de Ciencias Biológicas, Instituto Politécnico Nacional, Ciudad de México 07738, Mexico

**Keywords:** glucose oxidase, electrospraying, encapsulation, freeze-drying, oven drying, critical point drying

## Abstract

Glucose oxidase (GOX) is widely used in bakery applications to improve dough rheology and bread quality. However, its direct addition to formulations limits its functionality due to premature enzymatic activity. This study used electrospraying to encapsulate GOX using chia mucilage and sodium alginate as biopolymeric wall materials. Three drying methods—critical point drying (CPD), Lyophilization/freeze-drying (LC), and oven drying (OD)—were compared to evaluate their impact on encapsulation efficiency (EE), enzymatic activity retention, and microstructural integrity. Our findings reveal that CPD preserved the porous structure of the microcapsules, minimizing enzymatic leakage and yielding the highest EE (70%). In contrast, LC induced ice crystal formation, disrupting the polymer network and leading to a moderate EE (27.43%), whereas OD resulted in extensive capsule shrinkage, causing significant enzyme loss (57.1%). The release kinetics of GOX during mixing were best described by the Korsmeyer–Peppas model (R^2^ = 0.999), indicating a non-Fickian diffusion mechanism influenced by polymer relaxation. These results demonstrate that drying technique selection plays a crucial role in encapsulated enzymes’ stability and release behavior, providing new insights for optimizing enzyme delivery in bakery applications.

## 1. Introduction

Glucose oxidase is an enzyme with high specificity and efficiency, and with an increasing use for different industrial processes due to its high activity rate [1]. This enzyme is also receiving great attention in the food industry due to its improving properties in baking products [2]. GOX is also used in the production of biosensors [3], and as a food improver for the baking industry [4,5,6], replacing chemical additives [7]. The use of biological substitutes over chemical additives helps to address consumer demands for natural products by providing some additional benefits in foods as well as to consumers. However, in baking, the enzyme is used directly in the food matrix, presenting its maximum activity in the first 6 min of mixing without significant subsequent changes [8], affecting the volume and final quality of the bread [9]. These limitations could be overcome by using encapsulation methodologies. In this context, the use of electrohydrodynamic atomization or electrospraying is a promising method since, in addition to being a technique that is used at room temperature, it generates monodisperse particles [10]. In this sense, some enzymes such as lysozymes, proteases, glucose oxidase, and xylanases have been very efficiently encapsulated [11,12,13,14], making it a promising technique in the encapsulation of enzymes. However, once the capsules are obtained, they start to present physical and chemical instabilities such as aggregation, fission of the particles or hydrolysis of the coating polymeric materials [15], as well as a release of the active compound into the medium. These instabilities make it necessary to include a wall material stabilization process, drying being the most used one. Chia (*Salvia hispanica* L.) is a natural source of mucilage, a polysaccharide hydrocolloid known for its high water retention capacity, gel-forming ability, and film-forming properties. The mucilage extracted from chia seeds has been extensively studied in the food industry due to its biodegradable nature, biocompatibility, and ability to form stable encapsulation matrices [13]. Its high molecular weight and viscoelastic properties allow it to act as a protective barrier, reducing enzymatic degradation and enhancing controlled release in encapsulation systems [16]. Specifically, its capacity to retain moisture and provide structural integrity under different drying conditions makes it a promising alternative to synthetic polymers for enzyme delivery [17]. These characteristics make chia mucilage an ideal material for glucose oxidase encapsulation, ensuring enzymatic stability during storage and controlled release during the baking process.

The drying technique is a critical step of the encapsulation process, as it can influence the yield, the encapsulation efficiency (EE), the porosity, stability, and particle size [18]; oven drying and freeze-drying are the most frequently used, and the critical point drying technique is used less frequently. Oven drying uses heat to evaporate moisture from the sample, leaving dry powder. Lyophilization does not use high-temperature heating but sublimated water under a vacuum condition, which is helpful for heat-sensitive materials. Critical point drying is based on a hydrated organic sample (hydrogel polymers, starch) or biological sample (cells, tissues, blood, parasites, among others) being previously treated with different solvents and then the fluid is displaced in order to maintain the initial structure of the hydrated state [19]. Drying techniques are crucial in preserving enzymatic activity as they can induce structural modifications in encapsulation matrices that affect enzyme stability and release. Several studies have shown that convection drying can lead to enzyme denaturation due to prolonged exposure to heat. For example, Aaqil et al. (2023) [20] reported that polyphenol-converting enzymes are inactivated by heat during drying during black tea processing, which directly affects the product’s final quality. Similarly, research by Zuo et al. (2024) [21] highlighted that post-harvest drying methods significantly influence enzymatic activity and the retention of bioactive compounds in Styphnolobium japonicum flowers. Their findings suggest that high-temperature air drying rapidly inactivates degradative enzymes, whereas freeze-drying preserves enzyme functionality but allows prolonged enzymatic reactions, which may lead to undesirable metabolic changes.

On the other hand, in polysaccharide-based encapsulation systems, the selection of the drying method is critical for enzyme retention. Chen et al. (2024) [22] demonstrated that freeze-drying maintains higher enzymatic activity due to minimal thermal stress. In contrast, hot air drying and infrared drying promote molecular aggregation, which reduces enzymatic degradation but potentially disrupts the integrity of the polymer network. Furthermore, Guan et al. (2024) [23] demonstrated that microwave-assisted drying effectively reduces enzymatic activity while improving the structural properties of dried food materials, highlighting the need for a balance between enzymatic stability and matrix integrity.

However, after the different dehydration methods are applied, the morphostructural characteristics of the capsules, and the retention and protection of the active compound may be affected, which is associated with fractures, cracks, porosity, and the degree of integrity of the samples. These changes in the structure of capsules, and mainly on their surface texture, can be followed by scanning electron microscopy (SEM) and analyzed through image analysis, where the space disposition of the pixels’ grey levels are attributes that allow the roughness and softness of the capsule image surfaces to be quantified [24]. Based on previous studies, we hypothesize that selecting the drying method significantly influences the encapsulated enzyme’s structural and functional properties. Specifically, we expect critical point drying (CPD) to provide the highest encapsulation efficiency and enzymatic activity retention due to its ability to preserve microcapsule morphology. At the same time, freeze-drying (FD) may lead to partial structural alterations, and oven drying (OD) could result in severe capsule deformation and enzymatic inactivation. Therefore, the primary objective of this study is to evaluate the effect of different drying methods (critical point drying, freeze-drying, and oven drying) on the encapsulation efficiency, enzymatic activity retention, and morphological characteristics of glucose oxidase (GOX) microcapsules produced by electrospraying. Additionally, this study assesses the impact of stress conditions, such as mixing and fermentation, on the stability and release profile of the encapsulated enzyme, aiming to optimize its application in bakery food products.

This study aims to provide insights into optimizing enzyme encapsulation techniques for food and biotechnological applications, offering a comparative evaluation of drying methods to determine the most suitable approach for preserving enzymatic functionality.

## 2. Materials and Methods

### 2.1. Materials

Glucose oxidase from *Aspergillus niger* (19,290 U/g, Sigma-Aldrich, St. Louis, MO, USA; 9001-37-0), Chia seeds (*Salvia hispanica* L.) donated from local producers at La Palma, Nopala de Villagran (Hidalgo, Mexico), CaCl_2_ (JT. Baker, Avantor Performance Materials, Center Valley, PA, USA; 1313-01) 1313-01) and sodium alginate (Sigma-Aldrich, St. Louis, MO, USA; 9005-38-3) were used.

### 2.2. Methods

#### 2.2.1. Enzyme Encapsulation

The production of encapsulated glucose oxidase (GOX) at a concentration of 20 U/mL was carried out according to the methodology reported by Rentería-Ortega et al. (2021) [13]. The encapsulation solution was prepared by mixing chia mucilage and sodium alginate (1:1 *w*/*v*) in distilled water; it was homogenized under controlled stirring (800 rpm) for 30 min/25 °C, then the enzyme (20U) dissolved in 1 mL of acetate buffer (pH 5.1) was added. It was allowed to homogenize for an additional 30 min until a homogeneous mixture was obtained (surface tension = 65.67 ± 1.53 dynes/cm, viscosity 0.3809 ± 0.012 Pa·s, density = 0.99 ± 0.44 g/mL).

Encapsulation was performed using electrohydrodynamic atomization (EDHA) method under the following conditions: voltage 17 kV, the negative electrode connected to an aluminum vessel (12 cm diameter) filled with 2% (*w*/*v*) CaCl_2_ solution (JT. Baker, 1313-01), the distance between the tip of the needle (capillary) and the surface of the gelling solution (2% *w*/*v* CaCl_2_) was 5.5 cm, flow rate 2 mL/h and capillary diameter 0.6 mm (ID). Finally, the obtained capsules were collected from the gelling solution with a sieve (0.149 mm). They were analyzed before and after dehydration. The selection of encapsulation parameters was based on preliminary optimization trials and literature reports to ensure high encapsulation efficiency and controlled release.

#### 2.2.2. Enzymatic Activity (AE) and Encapsulation Efficiency (*EE*)

The enzymatic activity (AE) of glucose oxidase (GOX) was determined by measuring the consumption of oxygen during the oxidation of β-D-glucose, following the methodology described by Rentería-Ortega et al. (2021) [13]. A mixture of 1 mL of a 2% D-glucose solution (Sigma-Aldrich 50-99-7), 0.1 mL of a 2% ortho-dianisidine solution (Sigma-Aldrich 119-90-4), 0.1 mL of horseradish peroxidase solution at 60 U/mL (Sigma-Aldrich 9003-99-0), and 0.8 mL of glycerol (Sigma-Aldrich 200-289-5) was prepared. All solutions were prepared in 0.2 M Tris buffer (pH 7.0). Then, 10 µL of the sample (enzymatic solutions, hydrated capsules, dehydrated capsules, or capsules treated under different stress conditions or without stress) was added. The mixture was incubated at 30 °C for 30 min, and the reaction was stopped by adding 2 mL of 5M HCl. The absorbance of the mixture was measured at 525 nm using a spectrophotometer (Genesys 10 uv, Thermo Fisher Scientific, Madison, WI, USA). The obtained absorbance values were converted into glucose oxidase activity units (U/mL) using a standard curve of glucose oxidase (Sigma-Aldrich 9001-37-0) prepared at 0 to 20 U/mL. Regarding the determination of the encapsulation efficiency of GOX (Equation (1)), the CM-AlgNa hydrogel capsules containing the enzyme (GOX) were collected from the gelling solution (CaCl_2_) immediately after their preparation. In all cases, the measurements were performed in triplicate.

(1) $$EE\left(\%\right)=\left(\frac{Encapsulated\;enzymes\;activity}{Enzymatic\;activity\;in\;initial\;solution}\right)\times 100$$

### 2.3. Dehydration Processes

#### 2.3.1. Lyophilization

The capsules were distributed in a petri dish, frozen at −80 °C, and lyophilized in a Freeze Dryer; ECO-FD10PT (LabTech, Sorisole, Bergamo, Italy) according to the methodology reported by Czyzewska and Trusek, (2023) [25]. The procedure began with an initial freezing of the samples for 2 h at a temperature of −80 °C. Subsequently, the frozen samples were transferred to the lyophilizer. The lyophilization process was carried out by maintaining a temperature of −65.8 °C for 24 h and applying a vacuum pressure of 0.00001 Pa in order to minimize damage to the structure of the capsules and preserve the functionality of the encapsulated glucose oxidase.

#### 2.3.2. Convection Oven

The dehydration of the capsules by convection oven was carried out according to what was reported by Silva, 2019 [26] with some modifications. The capsules were subjected to convective drying at 30 °C for 8 h in a forced-air oven (Binder, Tuttlingen, Germany; 9010-0187) to remove residual moisture while preserving enzymatic activity. The drying temperature and duration were selected based on preliminary trials and literature reports to balance effective water removal and enzyme stability.

#### 2.3.3. Critical Point Drying

**Figure 1:**
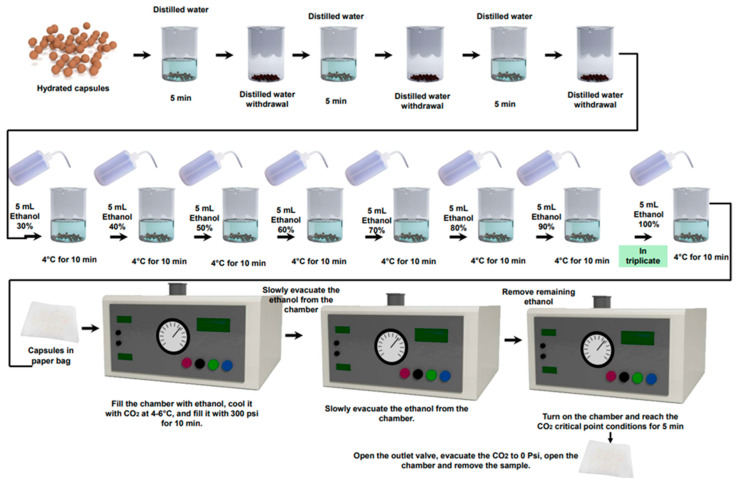
Critical point drying sequence. The process involves progressive dehydration using ethanol solutions of increasing concentration, followed by critical point drying with CO_2_. The red button controls the CO_2_ filling, the black button regulates pressure, the blue button adjusts temperature, and the green button releases residual CO_2_ after drying.

The critical point drying was carried out according to the methodology reported by Quispe (2020) [27]. In summary, the capsules were washed with distilled water to remove the gelling solution (CaCl_2_). Then, they were immersed in ethanol at different concentrations (3–100%) at 4 °C for 10 min at each concentration; afterwards, the samples were introduced in the critical point drying chamber of the equipment (K850, Quorum, Quorum Technologies, Laughton, East Sussex, UK). For a drying cycle, the chamber was filled with solvent (anhydrous ethanol), and cooled at slow flow to reach from 4 to 6 °C, by using liquid CO_2_ as the cooling media. Liquid CO_2_ was passed through the chamber until the gauge read 300 psi, displacing the solvent from the sample. After solvent displacement, liquid CO_2_ was purged by heating and pressurizing the chamber to approximately 34 °C and 1200 psi, above the critical point of CO_2_ (31.1 °C, 1071 psi) [28]. When these conditions were reached, the temperature was turned off and the samples were let to stand (incubated) for 5 min; finally, the pressure was reduced by opening the CO_2_ outlet valve until the pressure gauge reached 0 psi; the chamber was opened, and the dry encapsulates were removed (Figure 1). The experiments were carried out in triplicate.

### 2.4. Moisture Content

The percentage of moisture in the capsules was determined using a moisture analyzer (Ohaus Corp., Pine Brook, NJ, USA). The wet samples were placed in the device following the supplier’s specifications. The minimum weight of the sample was 0.5 g, with a rapid temperature program at a drying temperature of 105 °C for 10 min. Dried samples were also checked for moisture content, and in this case, the same methodology for wet samples was used, only reducing the drying time to 5.5 min, instead of 10 min. The average of the moisture content was calculated using 3 independent samples per drying method.

### 2.5. Micromorphology Analysis

#### 2.5.1. Confocal Laser Scanning Microscopy (CLSM) Analysis

The micromorphology of capsules (GOX CM-AlgNa) was observed in a confocal multiphoton microscopy LSM 710 NLO (Carl Zeiss, Oberkochen, Germany). The staining for the enzyme and the coating material was performed prior to the formation of the capsules. With respect to GOX, the methodology reported by Zhang et al. (2016) [29] was used with some modifications. Briefly, 0.1 mL of Fluorescein 5 (6)-isothiocyanate (FITC) (Sigma-Aldrich 46950) dye solution was added to 1 mL of carbonate buffer at pH 9.5; then, the enzyme was directly incorporated at a concentration of 20U. Subsequently, the sample was stored at 5 °C for 2 h for coupling the enzyme with FITC. On the other hand, the polymer matrix was stained with calcofluor white stain (Sigma-Aldrich 18909, 12171500), at a concentration of 2%. After the coupling time had elapsed, the enzyme was mixed with the coating material and stirred for 20 min at 800 rpm. The excitation and emission wavelengths used for FITC were 488 nm and 507–581, while for calcofluor, they were 405 nm and 410–458, using a 20×/0.8 objective and 1024 × 1024 resolution. The identification, delimitation, and 3D reconstruction of encapsulates were obtained and analyzed using the confocal microscope software (ZEN 2.3 SP1, Carl Zeiss, Germany).

#### 2.5.2. Scanning Electron Microscopy (SEM)

The overall morphology and texture parameters of the capsules were studied using SEM. Before observing by microscopy, samples were coated with gold using a sputter coater (SPI supplies, West Chester, PA, USA) and examined with a scanning electron microscope (Hitachi, SU3500 I, Tokyo, Japan) at 15.0 kV. From each sample, twelve images were studied. Initially, the SEM images in RGB format were converted to grayscale using the ImageJ software (v. 1.46, National Institute of Health, Bethesda, MD, USA), employing its plugins: MapFractalCount and Gray Level Correlation Matrices (GLCMs). Texture image analysis was done through fractal dimension (FD) and entropy (ET) parameters. According to Arredondo-Tamayo et al. (2023) [30], FD values close to 2 represent smooth surfaces, while higher ET values are related to heterogeneous surfaces.

### 2.6. Effect of Stress Conditions

#### 2.6.1. Mixing (Shear Stress)

The kinetic release of glucose oxidase (GOX) was evaluated at three time points (4, 6, and 8 min) and selected based on their relevance to the dough mixing process in bakery applications. These time intervals were chosen to mimic the enzymatic activity during the first stages of dough development, where GOX interacts with gluten proteins and oxidative reactions occur. Previous studies have demonstrated that the enzymatic effect of GOX is most pronounced within this timeframe, influencing dough rheology and gas retention properties.

Regarding the above, the encapsulated enzymes (1 g), obtained by EDHA and dehydrated by freeze-drying, oven, and critical point, were subjected to stress conditions simulating the mixing stage of the breadbaking process, according to the methodology of Rentería-Ortega et al. (2021) [13]. The capsules were mixed in a 2% D-glucose solution (Sigma-Aldrich 50-99-7) in a 1:1 ratio (dehydrated microcapsules–D-glucose solution). They were subsequently subjected to mechanical stress on a magnetic stirring plate (Benchmark Scientific, Sayreville, NJ, USA; H3770-HS) at a constant speed of 58 rpm/25 °C for 4, 6, and 8 min. The residual enzymatic activity (enzyme that remained inside the capsule) was subsequently evaluated according to Section 2.3.

#### 2.6.2. Fermentation Times (pH Stress)

The selection of fermentation conditions and pH stress parameters in this study was based on our previous research [13]; these conditions were based on their relevance to the intended application of GOX in baking. Studies have demonstrated that glucose oxidase exhibits optimal catalytic activity at pH 5.5–6.5 and temperatures between 30 °C and 40 °C. According to the above, encapsulated glucose oxidase (GOX) was evaluated under simulated bread-making conditions. Our prior findings indicated that GOX release from chia mucilage–sodium alginate capsules was influenced by pH, with lower pH values stabilizing the enzyme within the encapsulation matrix. In comparison, higher pH values facilitated faster enzymatic release. Therefore, in this study, pH values of 5, 6, and 7, along with fermentation times of 0, 80, 120, and 140 min, were selected to assess the impact of pH variations on enzyme activity over time. To emulate the fermentation stage, GOX-CMAlgNa capsules (6 g) were suspended in a 2% glucose solution (1:1 ratio) prepared in 0.2 M Tris Buffer at different pHs (5, 6, and 7), then left to stand in a temperature-controlled bath (ICB, BMF100917, Mexico) for different times (0, 80, 120, and 140 min) at 30 °C. Finally, the residual enzymatic activity (GOX inside the capsule) was evaluated, according to the procedure previously described in Section 2.2.2.

#### 2.6.3. In Vitro GOX Release Study and Kinetics

The GOX release study was conducted under simulated physiological conditions according to Section 2.6.1 and Section 2.6.2, under stress conditions involving mixing, pH, and time. The data were fitted to the Korsmeyer–Peppas model [31], which assesses the release rate where matrix erosion or dissolution occurs. It is expressed as the mass fraction released according to the kinetic constants “n” and K (Equation (2)). Constants are determined by plotting data and evaluating the linearity of the graph using the determination coefficient (R^2^ value).

(2) $$\frac{Mt}{M\alpha }={Kt}^{n}$$

where Mt is the absolute amount of GOX released at time t, Mα is the total amount of GOX released at infinite time, K is a constant that incorporates the structural and geometric characteristics of the delivery system, and n is a diffusional exponent that depends on the release mechanism and considers the mode of transport.

### 2.7. Experimental Design and Statistical Analyses

Regarding encapsulation efficiency, data were processed by multiple regression analysis, and the statistical significance (*p* ≤ 0.005) of the model and its variables was reported. In contrast, the measurement of encapsulation efficiency was statistically analyzed using SigmaPlot V.12.0 software (Systat Software Inc., San Jose, CA, USA). Each experiment was conducted in triplicate (n = 3), and results are expressed as mean ± standard deviation (SD). Statistical analysis was performed using one-way ANOVA, and differences between means were analyzed using Tukey’s post hoc test (*p* < 0.05).

A statistical design was used for capsules subjected to pH and time stress (fermentation). The rotatable composite was centered on its faces, with five repetitions of the central point and duplicates at the other points. The response was the residual enzymatic activity. The data were processed using Design Expert V9 software (Stat-Ease Inc., Minneapolis MN, USA).

## 3. Results and Discussion

### 3.1. Effect of the Drying Method on Encapsulation Efficiency

**Figure 2:**
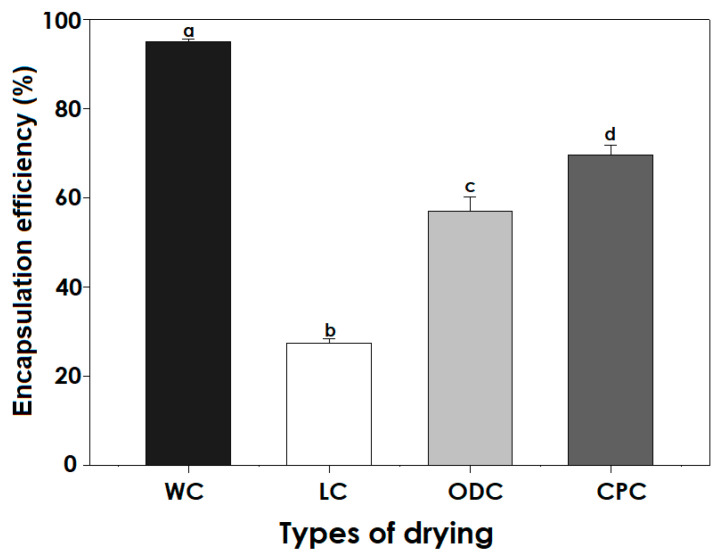
Effect of the drying method on encapsulation enzymatic activity. WC, Wet capsules; LC, lyophilized capsules; ODC, oven-dried capsules; CPC, critical point. Different letters over bars (a–d) indicate significant differences among drying methods (*p* < 0.05).

Encapsulation efficiency (EE) corresponds to the number of enzyme units inside the capsules, from the total initially added. A high EE indicates that GOX is adequately encapsulated in the matrix, which improves stability against oxidation during storage and processing. Figure 2 shows the encapsulation efficiency of the samples, where those without drying (WC) obtained values higher than 92 ± 1.34%, while dehydrated samples presented different enzyme retention capacities. In the case of the lyophilized material (LC), the lowest enzymatic retention values were obtained, followed by those materials dried in the oven (ODC). At the same time, the highest EE was obtained by the critical point drying method, keeping more than half compared to non-dried samples (WC).

These differences can be attributed to the structural integrity of the polymer network during the drying process. CPD has been reported to preserve the porous structure of encapsulation matrices, which prevents enzymatic leakage during drying [32]. In contrast, LF involves the formation of ice crystals, which can disrupt the encapsulation matrix, leading to reduced EE values [33]. Furthermore, the higher EE observed in CPD can be explained by the mild drying conditions, which minimize enzyme inactivation and polymer shrinkage. Similar findings were reported by Silva et al. (2019) [26], where enzymes encapsulated using CPD maintained higher bioactivity than other drying methods. These results suggest that CPD is a promising technique to preserve enzymatic activity in biopolymer-based encapsulation systems.

In addition to the above, the results obtained were similar to those reported by Alpizar-Reyes et al. (2020) [34] when using tamarind mucilage to encapsulate sesame seed oil, with values from 81.22 to 91.05%, the same as those established by Blandino et al. (2001) [35] in GOX capsules, and similar to the ones cited by Rentería-Ortega et al. (2021) [13] through electrohydrodynamic atomization. In addition to the above, Cetinkaya et al. (2020) [36] obtained encapsulation efficiency values higher than 94% for fish oil in Kafirina shells.

The high encapsulation efficiencies (EEs) observed in our work are probably related to the viscosity of the polymer matrix (0.3809 ± 0.012 Pa·s) and its rapid cross-linking due to the divalent cations of calcium chloride, which bind in the guluronate blocks (G blocks) present in sodium alginate. In this sense, solution viscosity minimizes the circulation of the active compound and the oscillation within the particles, causing a decrease in the migration of the GOX enzyme to the surface, as reported by De Campo et al. (2017) [37], while the cross-linking between mucilage, sodium alginate, and calcium ions on the surface of the drop results in the formation of wall barriers with lower internal water content, causing a high degree of cross-linking on the surface of gout [38]. The fact that 100% EE has not been achieved is because part of the enzyme found on the surface of the capsule is closely distributed in the porous matrix of the polymer, causing the surface enzyme to diffuse into the medium.

### 3.2. Effect of the Drying Method on the Moisture Content

**Figure 3:**
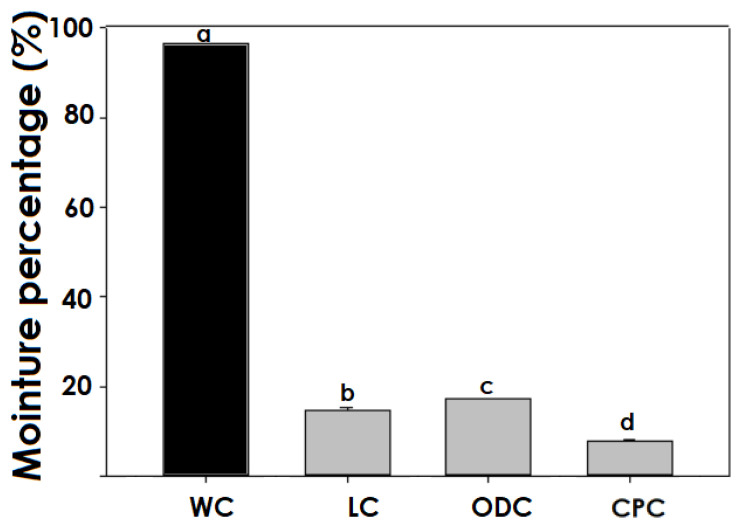
Percentage of moisture in capsules made by EDHA. WC, Wet capsules; LC, lyophilized capsules; ODC, oven-dried capsules; CPC, critical point. Different letters over bars (a–d) indicate statistically significant differences among drying methods (*p* < 0.05).

The moisture content of the capsules as obtained from the EHDA was very high (>95%) (Figure 3). Subsequently, when dehydrated by any of the methods (freeze-drying, oven, and critical point drying), the original values decreased to a minimum of 5% from 18.6%, depending on the drying technology applied. The microcapsules dried by freeze-drying showed the highest moisture content (18.6%), associated with the fact that rapid freezing caused contraction in the outer layer, leading to a smaller pore size that made mass transfer between the internal and external environment difficult.

On the other hand, lyophilization-dehydrated capsules present the lowest percentage of moisture compared to oven-dried, due to the sublimation of frozen water; in this regard, it has been stated that vapor diffusion increases with the porosity of the materials and that a fast freezing rate promotes the formation of small crystals with pores after freeze-sublimation [39], increasing the resistance to mass transfer and leading to a higher percentage of moisture. At the same time, the pressure gradient, as a driving force, affects the removal of moisture, enabling the contraction of the polymeric matrix, leaving voids within the capsule structure, as reported by Chan et al. (2011) [40], and developing undesirable shapes, uneven capsule size, and high porosity, characteristics related to a rapid release of the active compound. According to the above, Nowak and Jakubezyk (2020) [41] mention that in most cases, the percentage of humidity reaches a water loss greater than 95% in pharmaceutical and food products. However, these values will depend on the composition and chemical structure of the biopolymer to eliminate free and bound water, Espinosa et al. (2022) [42].

The differences observed in the oven drying, with respect to the critical point methodology, could be associated with the speed and degree of water migration. In the oven drying method, heat is transferred from the hot air to the surface of the capsules, promoting water evaporation. However, the shrinkage of the polymer matrix during oven drying reduces the pore size. It acts as a physical barrier limiting mass transfer and hinders moisture diffusion from the core to the outside of the capsule. This effect results in a higher residual moisture content than is observed in the critical point drying method, where water removal occurs in a more controlled and uniform manner. In this sense, Espinosa et al. (2022) [42] reported the formation of a surface crust in food drying by forced convection, which reduced the drying rate in the final stages of the process. On the other hand, Khater et al. (2024) [43] mention that an increase in temperature and hot air flow inside the drying chamber increases mass and heat transfer, leading to steeper drops in moisture content. An opposite effect was observed at low drying temperatures. These results agree with those reported by Ho et al. (2022) [18] in xanthone encapsulations by coacervation, who found that a temperature of 50 °C in oven drying resulted in high moisture content due to the low efficiency of heat and mass transfers.

Finally, critical point (CP) drying presented the highest humidity reduction among the evaluated methods, probably because, during the process, the fluid present in the capsules (H_2_O) is gradually replaced with an intermediate solvent (CO_2_) and then removed under critical point conditions, minimizing the surface tensions that usually occur in other drying methods, such as lyophilization or convection oven drying, which can generate contraction or partial closure of the pores. Critical point drying facilitates a more efficient mass transfer by maintaining the porous and open structure of the material during the process, allowing complete extraction of the fluid without leaving significant residual water and maintaining the initial structure of the hydrated state [44].

While critical point drying resulted in the most significant moisture reduction, these results were associated with the soft and elastic consistency of the capsules in the hydrated state. These characteristics are associated with the low cross-linking density of the biopolymers, as reported by Katime et al. (2004) [45].

The results indicate that the drying technique significantly influences the final moisture content of the encapsulated glucose oxidase (GOX) particles, with critical point drying (CPD) yielding the lowest moisture levels and oven drying (OD) the highest. However, it is important to note that this study focused on the direct impact of drying methods on moisture retention and did not evaluate the effects of environmental factors such as humidity and storage conditions.

Environmental factors, including relative humidity, temperature fluctuations, and exposure to oxygen, influence moisture equilibrium and could impact the long-term stability of encapsulated enzymes [46]. Previous studies have demonstrated that encapsulated biopolymers may experience moisture reabsorption when stored under high humidity conditions, which could alter their functional properties. Ahmetli et al. (2024) [47] reported that biopolymeric materials exposed to high humidity levels undergo swelling and structural relaxation, which may compromise the mechanical integrity of the encapsulated matrix and increase enzymatic degradation. These findings suggest that humidity control is critical in ensuring encapsulated bioactive compounds’ long-term stability. Future research should explore the influence of storage humidity on the moisture content and enzymatic stability of these encapsulates, particularly in food processing environments where fluctuations in ambient conditions are common. Additionally, assessing encapsulated powders’ water activity (aw) under controlled humidity chambers could provide further insights into their stability and shelf life.

### 3.3. Effect of Drying Method on Morphometric Parameters

The morphometric parameters of the capsules dried by the different methodologies are shown in Table 1. It was observed that the oven-dehydrated ones collapsed, forming a film, making it impossible to determine their morphological properties, which suggests that this method does not preserve the structural integrity of the capsules. The samples dried by this method exhibited severe structural deformation due to polymer shrinkage and humidity gradient effects. This behavior is consistent with the findings of Najjaa et al. (2020) [48], who reported that conventional oven drying induced significant matrix collapse and reduced the retention of bioactive compounds. These results highlight the importance of selecting an appropriate drying method to maintain encapsulation integrity and enzymatic stability.

On the other hand, the lyophilized material exhibited a larger diameter (544.4 ± 52.6 µm) and perimeter (1663.6 ± 204.9 µm), values significantly higher (*p* < 0.05) than those obtained for the encapsulates dried by critical point, where the diameter was 305.6 ± 53.0 µm and the perimeter was 1077.4 ± 190.6 µm. The increase in the size of the lyophilized encapsulates can be attributed to the fact that the lyophilization process, carried out at low temperatures and in the absence of liquid phases, minimizes contraction and allows the particles to retain a larger volume [49]. Regarding roundness, the encapsulates dried by critical point showed a value of 0.81 ± 0.06, significantly higher (*p* < 0.05) than that of the lyophilized encapsulates (0.66 ± 0.07). This indicates that the particles subjected to critical point drying exhibit a more spherical morphology, possibly due to the less invasive nature of this process, which allows for better preservation of the three-dimensional structure of the capsules [50]. The lyophilization process, although effective in maintaining the integrity of the capsule size, may induce surface deformations in the particles, affecting their sphericity [51].

### 3.4. Structural Analysis

#### 3.4.1. Confocal Laser Scanning Microscopy (CLSM)

**Figure 4:**
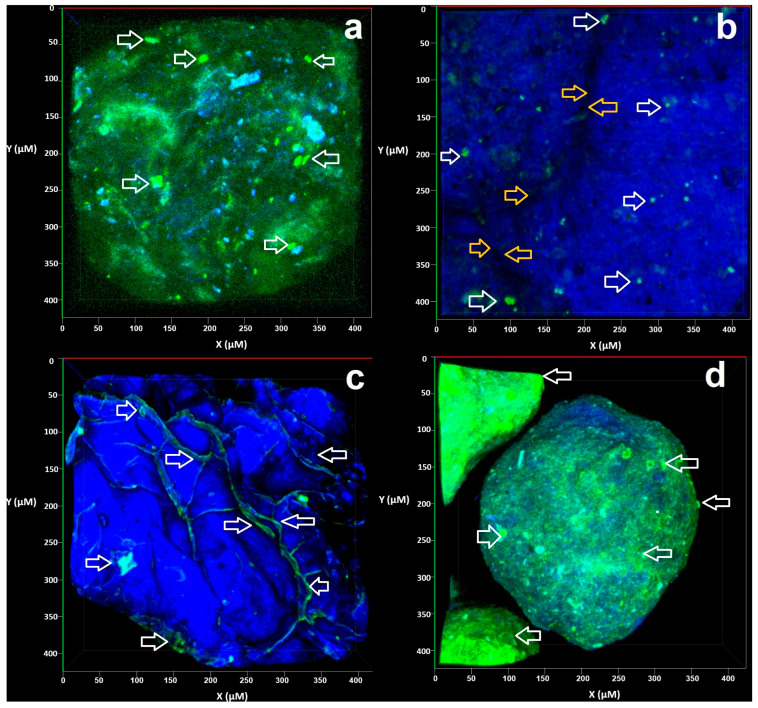
Confocal images of GOX encapsulated in CMAlgNa. (**a**) Hydrated encapsulates, (**b**) oven-dehydrated encapsulates, (**c**) lyophilized-dehydrated encapsulates, (**d**) critical point encapsulates. White arrows indicate the staining of the GOX enzyme with FITC, yellow arrows show the union between encapsulates, blue color shows calcofluor staining of the CMAlgNa biopolymer, while green color is related to fluorescein 5 (6)-isothiocyanate (FITC) staining protein.

Confocal laser scanning microscopy (CLSM) was used to observe the distribution of the enzyme in the polymeric matrix. The experimental results show a distribution of GOX (green color) embedded in the chia mucilage–sodium alginate (CM-AlgNa) matrix (blue color). According to the above, Figure 4 shows the encapsulates hydrated and dried by the different methods (oven drying, lyophilized, and critical point drying).

In Figure 4a (hydrated capsules), particles maintained their shape, presenting a more intense green-yellow color, demonstrating that the active compound was embedded in the polymer matrix. In Figure 4b (oven-dried capsules), the particles collapsed, losing their shape, because they are soft structures with high moisture content, where the surface tension forces associated with the exit of water caused structural damage; on the other hand, the evaporation of water from the particle’s interior reached a limit where the capsule collapsed.

Regarding the above, despite the collapse of the structure, the micrographs show the presence of the enzyme. In this sense, Aniesrani-Delfiy et al. (2015) [52] obtained similar morphologies using Arabic gum as an encapsulating material. On the other hand, the lyophilized particles presented a superficial distribution of the enzyme, a behavior associated with the sublimation of the water crystals that caused the contraction of the hydrogel matrices, leaving voids. This resulted in irregular, porous, and rough surfaces, as shown in Figure 4c. In addition to the above, Cengel et al. (2011) [53] attribute this behavior to the molecular displacement from the area of highest concentration to that of the lowest concentration. In other work, Callewaert et al. (2007) [54] reported that when they used this technique in alginate-based encapsulations, the spheres shrank, and some showed breaks. Likewise, Rassis et al. (2002) [55], through this process, obtained collapsed structures when sublimation of water from the hydrogel matrix occurred, resulting in beads with irregular shapes.

Finally, in Figure 4d, the capsules show an intact structure, confirming that the critical point drying method preserves the morphology of the polymeric matrix. The green color, representative of the glucose oxidase (GOX) enzyme, is homogeneously distributed on the surface and inside of the particles. This distribution pattern suggests that the active compound (GOX) remains encapsulated within the matrix (CM-AlgNa), with no signs of significant loss during dehydration. The difference in the color intensity of Figure 4d compared to the other figures can be attributed to localized GOX accumulation or differences in the residual hydration of the polymer matrix.

#### 3.4.2. Scanning Electron Microscopy (SEM)

**Figure 5:**
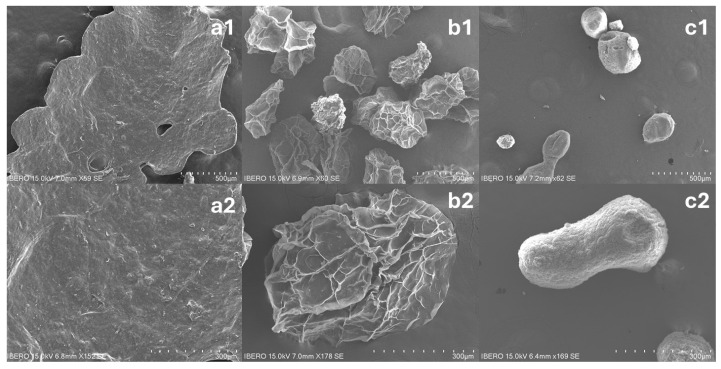
Scanning electron microscopy (SEM) images of (**a1**,**a2**) encapsulates dried by oven, (**b1**,**b2**) encapsulates dried by freeze-dried, and (**c1**,**c2**) encapsulates dried by critical point. The bar in the (**a1**–**c1**) images corresponds to 500 μm, and (**a2**–**c2**) corresponds to 300 μm.

The surface textures of the capsules dehydrated by three methods—oven drying (a1, a2), freeze-drying (b1, b2), and critical point drying (c1, c2)—are shown in Figure 5. The oven-dried capsules (a1, a2) exhibit an elongated and flat morphology with small agglomerates; while the polymeric matrix is partially preserved, the collapsed structure suggests significant disruption, making it unclear whether the enzyme remains entirely encapsulated or partially released. In contrast, the freeze-dried capsules (b1, b2) maintain their shape but an apparent shrinkage is observed, with grooves formed during dehydration; thus, these structural disruptions could indicate potential pathways for enzyme release, although this cannot be directly confirmed from the image alone. Lastly, the critical point-dried capsules (c1, c2) retain a spherical and uniform structure, with minimal deformation and no visible grooves, demonstrating that this method better preserves the structural integrity of the samples. This suggests that critical point drying is the most efficient method for preserving the original morphology.

From SEM micrographs, texture image analysis was performed using fractal dimension (FD) and entropy (ET) parameters.

Fractal dimension reflects the surface complexity of the encapsulates. Critical point-dried capsules present the highest value of fractal dimension (2.66 ± 0.06), indicating a more complex structure compared to freeze-dried (2.53 ± 0.06) and oven-dried (2.61 ± 0.04) samples. Different letters (a, b, c) indicate that the differences between the drying methods are significant (*p* < 0.05), suggesting that the drying method significantly affects the surface texture of the encapsulates. Entropy measures textures’ heterogeneity, and values of 7.79 ± 0.35 for oven drying, 7.65 ± 0.61 for freeze-drying, and 7.66 ± 0.59 for critical point drying were obtained. This indicates that although the drying methods generate differences in surface morphology, the texture heterogeneity does not vary significantly. According to Arredondo-Tamayo et al. (2023) [30], FD values close to 2 represent smooth surfaces, while the higher ET values are related to heterogeneous surfaces.

The fractal dimension results shown in Table 2 demonstrated that the capsules dried by critical point had the largest values; thus, the samples presented rough surfaces compared to the others. Samples dried by freeze-drying showed the lowest values, representing a material with smooth surfaces. Entropy (ET) values, which measure surface heterogeneity, did not differ significantly between drying methods (*p* > 0.05). However, the observed FD values align with previous findings by Arredondo-Tamayo et al. (2023) [30] on gellan gum films with eggshell nanoparticles with values around 2.51–2.64, that CPD-treated capsules exhibited higher structural complexity, which correlates with improved functional properties. The higher roughness in CPD-dried capsules may contribute to a more controlled and sustained enzyme release, avoiding burst effects commonly observed in softer freeze-dried structures.

### 3.5. Effect of Stress Conditions (Mixing, Fermentation) on the Residual Enzymatic Activity

#### 3.5.1. Mixing

**Figure 6:**
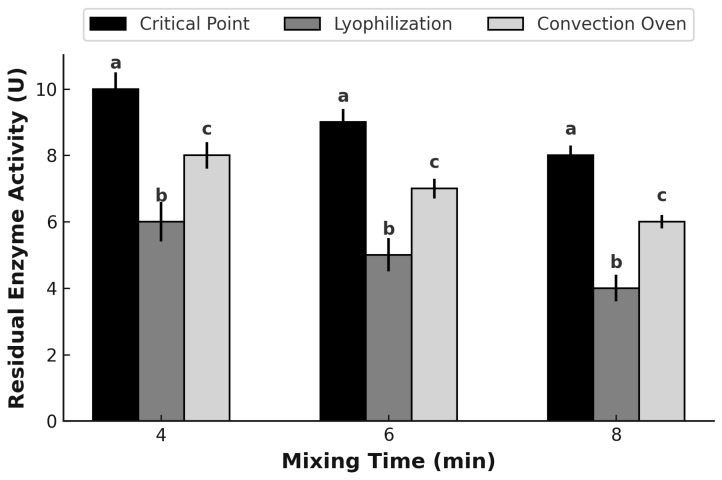
Effect of drying methods and mixing time on residual enzyme activity. Different letters over the bars (a–c) indicate statistically significant differences among drying methods for the same mixing time (*p* < 0.05).

Figure 6 illustrates the residual enzymatic activity of glucose oxidase (GOX) after being subjected to different dehydration methods (critical point drying, freeze-drying, and convection oven drying) and different mixing times (4, 6, and 8 min). The results reveal significant differences in the enzymatic activity depending on the dehydration method and mixing time, underlining the fundamental role of the drying process in preserving enzyme functionality. Among the methods evaluated, critical point drying demonstrated the highest residual enzymatic activity at all mixing times, reaching its peak at 4 min. This suggests that critical point drying is the most effective method for preserving enzymatic activity. This superior performance is likely due to the absence of surface tension and structural deformation commonly observed in other dehydration techniques, such as freeze-drying and convection drying. Furthermore, critical point drying maintains the integrity of the polymeric matrix, minimizing enzyme loss and preserving its functional properties.

These results are similar to those reported by Wang et al. (2021) [56], who cited that critical point drying effectively preserves the structural integrity of enzymes by minimizing thermal and mechanical stresses while preventing structural collapse of the encapsulation matrix. In addition, Rao et al. (2019) [57] emphasized that this method can maintain the structure and functionality of proteins sensitive to thermal changes.

In contrast, lyophilization, despite being a widely used technique to preserve bioactive compounds, showed significantly lower enzymatic activity compared to critical point drying, particularly at longer mixing times. This reduced activity may be attributed to the freezing and sublimation processes inherent to freeze-drying, which may partially denature enzymes [58]. On the other hand, the convection oven drying method showed intermediate residual enzymatic activity. Although performed at a relatively low temperature (30 °C), this method was less aggressive than freeze-drying. However, it did not achieve the enzymatic preservation achieved by critical point drying.

#### 3.5.2. Fermentation Stage (pH Stress)

**Figure 7:**
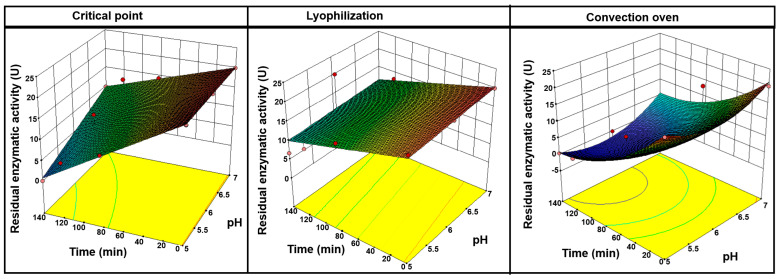
Effect of pH and resting time (t) on the residual enzymatic activity of the hydrogel capsules.

Regarding the residual enzymatic activity of encapsulated GOX subjected to various pH conditions during different resting times (Figure 7), a significant influence of both variables was observed (*p* ≤ 0.0001). In all cases and after 140 min, the enzymatic activity at pH 5 showed a progressive decrease as the resting time increased, reaching zero activity for critical point and convection oven methodology samples. For example, in critical point drying, the residual activity decreased from 20 U to 0 U at pH 5, and from 20 U to 10 U at pH 7. A similar behavior was observed in convection oven drying, where the residual activity at pH 7 decreased from 20 U to approximately 5 U, and at pH 5, it fell from 20 U to 0 U. These results suggest that pH does not have a significant effect at initial times, and the resting time has a predominant impact on the loss of enzymatic activity, especially under acidic pH conditions. This is attributed to the fact that biopolymers, such as chia mucilage and sodium alginate, are sensitive to specific pH ranges. Lyophilization followed a similar path to the other drying methodologies, but better preserved the enzyme activity.

At the initial stage (t = 0 min), statistical analysis (one-way ANOVA) confirmed that pH did not significantly affect enzymatic activity (*p* = 0.187). These results indicate no statistically significant differences (*p* > 0.05) among the tested pH conditions at the beginning of the process.

The absence of a significant pH effect at the initial stage (t = 0 min) can be attributed to the intrinsic stability of glucose oxidase over a relatively broad pH range. Previous studies have demonstrated that glucose oxidase retains significant activity within a pH range of 6.0 to 9.0 over short periods [59]. Similarly, Gao et al. (2012) reported that glucose oxidase exhibited stable catalytic activity across a wide pH range in aqueous environments, with only minor fluctuations in its efficiency. However, as time progresses, gluconic acid production during glucose oxidation may lead to a gradual pH decrease, which could negatively impact enzymatic activity [60]. This aligns with the findings of Wong et al. (2008) [2], who observed that while GOX maintains its structure in mildly acidic and neutral conditions, prolonged exposure to acidic environments can induce conformational changes that reduce its catalytic efficiency. These findings align with our results, indicating that pH alone does not play a decisive role in the initial enzymatic activity but becomes a more relevant factor over time, particularly due to the accumulation of reaction products that alter the medium’s chemical properties.

The above-mentioned results could be attributed to forming different matrix structures depending drying conditions, and releasing the enzyme as a function of the applied stress. In this sense, Ahmadi and Rodríguez (2024) [61] reported that less aggressive drying techniques, such as critical point drying, better preserve the capsule structure, increasing the encapsulation efficiency and the release of the encapsulated compound. This creates a denser matrix structure, resulting in better retention of active compounds and allowing more effective release in controlled environments. In this sense, Lláčka et al. (2023) [62] point out that the use of supercritical CO_2_ removes the solvent at lower temperatures and with less mechanical stress on the capsules, benefiting the stability of the encapsulated enzymes, removing water without passing through the liquid phase, thus avoiding surface tension that could induce structural collapses. In the case of convection oven drying, the decrease in enzymatic activity (EA) over time and under pH 5 conditions can be attributed to changes in the surface tension of the matrix caused by the removal of water, inducing the formation of larger or less ordered pores [63].

### 3.6. Kinetic Study of the In Vitro Release of GOX

#### Shear Stress (Mixing Stress)

**Figure 8:**
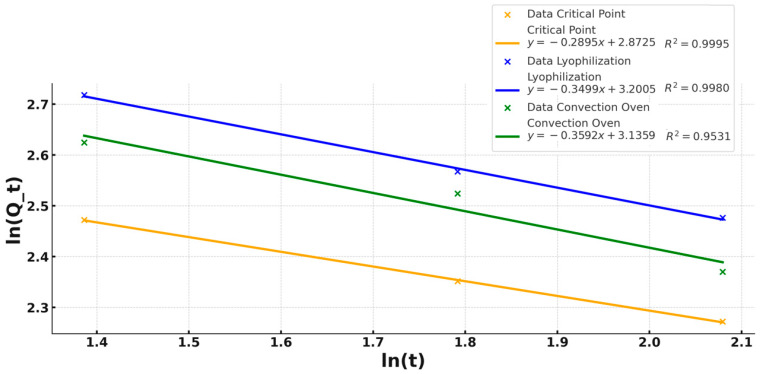
Kinetic model for the release of glucose oxidase under mixing stress. The curves show the Korsmeyer–Peppas models for the different drying methods (critical point drying, lyophilization, and convection oven drying), evaluating the residual enzymatic activity as a function of mixing time.

Various mathematical models were evaluated to describe the release kinetics of encapsulated glucose oxidase (GOX) during the mixing stage, including zero-order, first-order, and Higuchi models. However, these models showed correlation coefficients lower than the Korsmeyer–Peppas model. Among the tested models, the Korsmeyer–Peppas model (Figure 8) exhibited the best fit across all drying methods, with high correlation values (CPD: R^2^ = 0.999, LF: R^2^ = 0.998, OD: R^2^ = 0.953). This model accounts for diffusion and polymer relaxation mechanisms, particularly relevant for enzyme release from polysaccharide-based encapsulation matrices. Similar findings have been reported in previous studies where non-Fickian diffusion was identified as the dominant release mechanism in hydrocolloid-based encapsulation systems.

According to the above, this model demonstrated R^2^ values higher than 0.998 for critical point and lyophilization with all release exponents (n) < 0.5, indicating the mechanisms to describe the release of GOX from the polymeric matrix. According to the above, the shear stress (mixing) and the fitting lines in this graph indicated that the solvent diffusion is much larger than the relaxation process of the polymeric chain; that is, the kinetics of this phenomenon are characterized by diffusivity. This suggests that the release of the enzyme under drying methods, especially critical point drying and freeze-drying, may follow closely the behavior of an anomalous release model. In this sense, Ge et al. (2019) [64] report that the diffusion index n value affects the desorption mechanism. When 0 < n < 0.45, it is Fickian diffusion; when 0.45 < n < 0.89, it is non-Fickian diffusion or abnormal release; when n < 0.89, it is the skeleton dissolution mechanism, obtaining in all cases a diffusion index n less than 0.45, so the sustained release mechanism is Fickian diffusion.

## 4. Conclusions

This study demonstrated that the drying method significantly influences the encapsulation efficiency, enzymatic activity retention, and morphological integrity of glucose oxidase (GOX) encapsulated in chia mucilage–sodium alginate matrices. Critical point drying (CPD) provided the highest encapsulation efficiency and best-preserved enzymatic activity among the evaluated methods. At the same time, freeze-drying (FD) led to structural alterations, and oven drying (OD) caused severe capsule collapse. These findings highlight the importance of selecting an appropriate drying method to maintain enzyme stability in encapsulation systems.

Furthermore, the analysis of morphometric and structural parameters revealed that CPD preserves the capsules’ porous structure, favoring a controlled enzymatic release. In contrast, FD promotes excessive porosity, and OD results in matrix collapse. These structural differences directly impact the enzymatic performance, influencing potential applications in food systems where controlled release is required.

The novelty of this work lies in the comparative assessment of drying techniques for electrospray GOX encapsulates, providing insights into their impact on structural and functional properties. This study offers a reference for optimizing enzyme encapsulation for food and biotechnological applications, particularly in controlled-release systems. Additionally, these findings contribute to the growing field of bioencapsulation, reinforcing the importance of selecting suitable drying processes for preserving bioactivity in functional ingredients.

Future research should explore long-term storage stability under actual processing conditions and evaluate the performance of encapsulated GOX in specific food matrices. Further investigation is needed to refine drying conditions and improve capsule robustness for large-scale applications. Exploring the effect of additional encapsulation materials and their interactions with drying methods could further enhance enzymatic systems’ stability and controlled-release efficiency.

## Figures and Tables

**Table 1:** Morphometric and nanomechanical parameters of capsules.

Parameter	Feret Diameter (µm)	Perimeter (µm)	Roundness (-)
Oven drying	--------------------------	----------------------------	------------------------
Lyophilized Encapsulated	544.4 ± 52.6 ^a^	1663.6 ± 204.9 ^c^	0.66 ± 0.07 ^e^
Critical point encapsulations	305.6 ± 53.0 ^b^	1077.4 ± 190.6 ^d^	0.81 ± 0.06 ^f^

Different letters in the same column indicate that values are significantly different (*p* < 0.05). Dotted lines for oven drying data mean that samples could not be obtained by this technique.

**Table 2:** Texture parameters image analysis of each encapsulate studied.

	Oven	Freeze-Drying	Critical Point
**FD (-)**	2.61 ± 0.04 ^a^	2.53 ± 0.06 ^b^	2.66 ± 0.06 ^c^
**Entropy (-)**	7.79 ± 0.35 ^a^	7.65 ± 0.61 ^a^	7.66 ± 0.59 ^a^

FD: fractal dimension. Values presented are the average ± standard deviation. Different letters indicate that the values are significantly different (*p* < 0.05).

## Data Availability

The original contributions presented in the study are included in the article, further inquiries can be directed to the corresponding author.
